# Deletion of 4q28.3-31.23 in the background of multiple malformations with pulmonary hypertension

**DOI:** 10.1186/1755-8166-7-36

**Published:** 2014-06-05

**Authors:** Balazs Duga, Marta Czako, Katalin Komlosi, Kinga Hadzsiev, Katalin Torok, Katalin Sumegi, Peter Kisfali, Gyorgy Kosztolanyi, Bela Melegh

**Affiliations:** 1 Department of Medical Genetics, Clinical Centre, University of Pecs, Szigeti 12, Pecs H-7624, Hungary; 2 Szentágothai Research Centre, University of Pecs, Ifjusag 20, Pecs H-7624, Hungary; 3 Department of Pediatrics, Clinical Centre, University of Pecs, Jozsef Attila 7, Pecs H-7623, Hungary

**Keywords:** 4q28.3-31.23, Array CGH, Deletion, Complex malformation syndrome, Developmental delay, Pulmonary hypertension, Face dysmorphia, Vascular malformation of the lung

## Abstract

The 4q deletion syndrome shows a broad spectrum of clinical manifestations consisting of key features comprising growth failure, developmental delay, craniofacial dysmorphism, digital anomalies, and cardiac and skeletal defects. We have identified a de novo interstitial distal deletion in a 9 month-old girl with growth failure, developmental delay, ventricular septum defect in the subaortic region, patent foramen ovale and patent ductus arteriosus, vascular malformation of the lung, dysgenesis of the corpus callosum and craniofacial dysmorphism using array-comparative genomic hybridization. This de novo deletion is located at 4q28.3-31.23 (136,127,048 - 150,690,325), its size is 14.56 Mb, and contains 8 relevant genes (*PCDH18, SETD7, ELMOD2, IL15, GAB1, HHIP, SMAD1, NR3C2)* with possible contributions to the phenotype. Among other functions, a role in lung morphogenesis and tubulogenesis can be attributed to the deleted genes in our patient, which may explain the unique feature of vascular malformation of the lung leading to pulmonary hypertension. With the detailed molecular characterization of our case with 4q- syndrome we hope to contribute to the elucidation of the genetic spectrum of this disorder.

## Background

It has now been accepted in the literature that the 4q- syndrome comprises interstitial and terminal deletions of the long arm of chromosome 4 [1,2]. Essential features of this syndrome include mild craniofacial and digital dysmorphism, developmental delay, growth retardation, skeletal and cardiac anomalies, and autism spectrum disorder [2]. A broad spectrum of clinical manifestations has been observed partly due to the variability in the extent of the deletions and the possible additional contribution of other genetic rearrangements, such as unbalanced translocations [3]. Many reports of 4q- cases associated with large deletions could be detected by conventional chromosome analysis; however, in recent years the reports of submicroscopic chromosomal aberrations associated with 4q- phenotype increased as a result of the widespread diagnostic use of array comparative genomic hybridization (a-CGH) [3].

A classification of the 4q- deletions has been proposed by Strehle et al. based on the observation that deletions appear to occur in clusters along 4q [2]. As demonstrated in previous case reports, it is not uncommon that array CGH testing refines or corrects standard karyotyping. We present the case of a 9-month-old girl with a 14.56 Mb distal interstitial deletion of chromosome 4, more specifically in the region of 4q28.3-31.23. Reports of distal 4q interstitial deletions are quite rare. In addition to the key features seen in 4q- cases epilepsy, small kidneys, hyponatraemia, immunodeficiency and vascular anomaly of the lung vessels was also seen in our patient. There had been limited clinical phenotype and molecular correlation for congenital heart defects (CHDs) for this region until the routine use of aCGH, however, in a recent report of a child with a 4q32.3-q34.3 (chr4:167236114–178816031; hg18) deletion involving the TLL1 (Tolloid-like-1), HPGD (15-hydroxyprostaglandin dehydrogenase), and HAND2 (Heart and neural crest derivatives-expressed protein 2) genes, that are known to be involved in cardiac morphogenesis, the critical region responsible for CHDs seen in 4q deletion syndrome was narrowed down [4]. CHDs are reported to occur in about 60% of patients with 4q deletion syndrome in the presence or absence of HAND2 deletion, implying additional genes in 4q that could influence cardiac development. We discuss the possible role of eight deleted genes in the expression of the unique phenotype including the anomaly of the lung vessels seen in our patient. The precise molecular characterization of new cases with 4q- syndrome will aid to elucidate the genetic spectrum of this disorder.

## Case presentation

### Patient

The index patient was born as the first child to non-consanguineous healthy parents at 39^th^ week of gestation, with a birth weight of 2160 g by caesarean section, Apgar scores were 8/9. Because of heart murmur cardiologic examination was performed which revealed ventricular septum defect in the subaortic region, Patent Foramen Ovale and patent ductus arteriosus. Abdominal ultrasound showed smaller kidneys with normal structure and cranial ultrasound showed dysgenesis of the corpus callosum.

At the age of two month she was hospitalized due to feeding problems and hypoglycaemia (blood glucose value: 1,2 mmol/L). Severe dystrophy, muscle hypotonia and dysmorphic facial features including remarkable asymmetry of the face (hemihypertrophy of the left side), short right palpebral fissure, long eyelashes, asymmetry of the ears with a dysplastic, smaller and low-set right ear, short philtrum and high palate were detected. During hospitalization impairment of consciousness and short atonic periods were present, which were presumed to be convulsion equivalent, but the performed EEG did not indicate paroxysmal signs.

At the age of 3 months her congenital heart defect required surgical correction (closure of the ventricular septum defect, foramen ovale and ductus arteriosus). The results of laboratory tests showed no abnormality besides hyponatremia. After the heart operation there were no residual defects, the pulmonary pressure was normal. During the 4^th^ week of the postoperative period, a severe septic state developed. Because of an advanced AV-block a temporary pacemaker was needed. She was dismissed from the hospital and was regularly checked on by an outpatient cardiology clinic.

Four months following heart surgery she was admitted to the clinic again because of pulmonary hypertension. Radiological examinations (Computed Tomography (CT) and CT-angiography) were performed in order to exclude a possible pulmonary cause which revealed a lower arborisation of the pulmonary arteries supplying the left lower lobe. No branches were detected to the left upper lobe and a congenital anomaly was suggested, not excluding the possibility of a persistent non-recanalized thrombosis. Cardiomegaly was reported (primarily to the right part of the heart) with an enlarged pulmonary trunk as a result of elevated pulmonary arterial pressure. A left sided vena cava superior and also an atypically localized (left side) undefined venous vessel was present on the axial and the reconstructed CT slices. The vena cava inferior and also the hepatic veins were enlarged due to elevated venous pressure in the major blood circle. In the thorax cavity hydrothorax was found on the right side with consolidation on both sides and no normal lung parenchyma.

**Figure 1:**
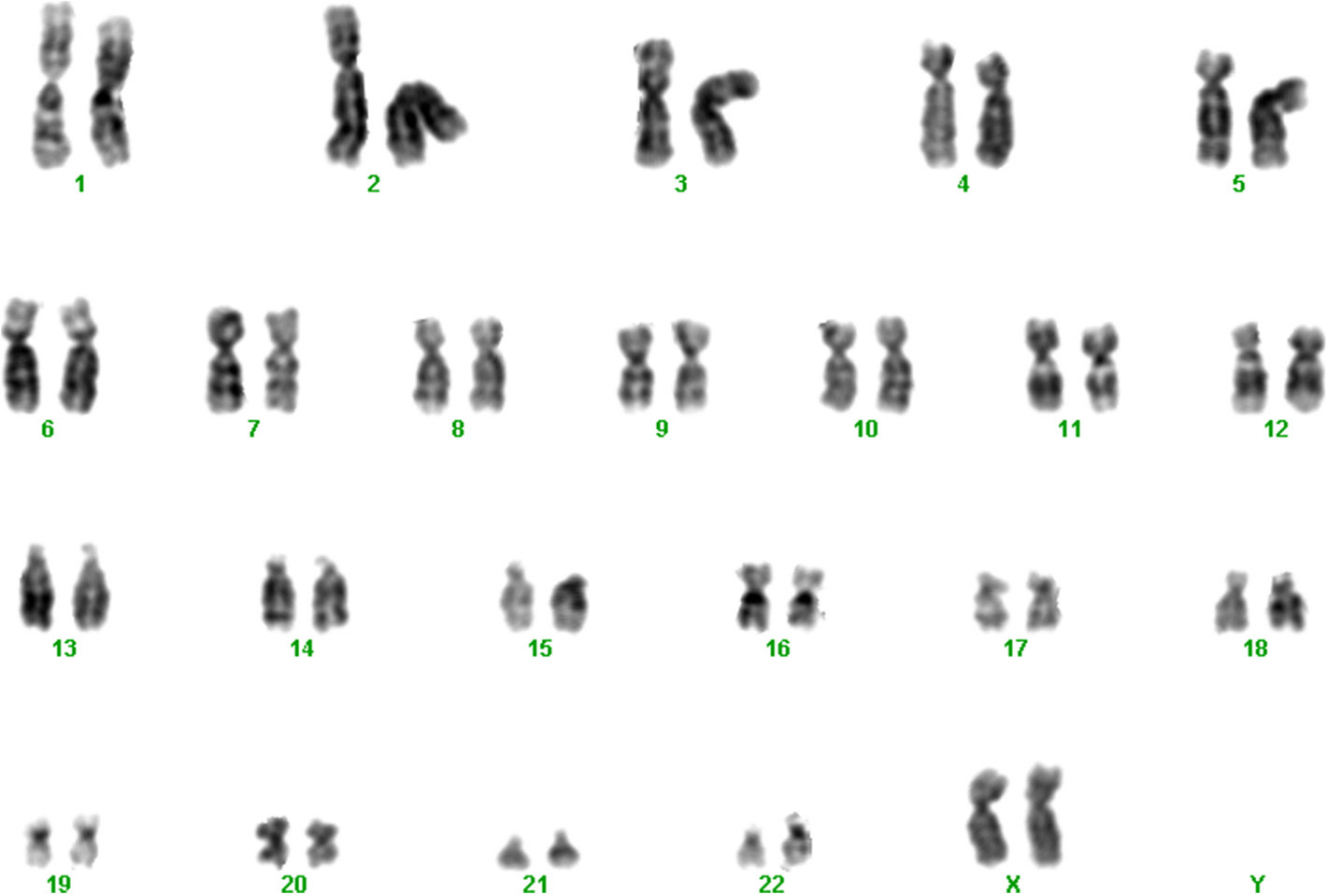
**Patient’s G-banded karyogram.** The banding resolution in this case remained about 400 bphs (bands per haploid set) which is below the minimum G-banding quality required for analysis of postnatal samples of patients with intellectual disability and/or dysmorphic features (550 bphs).

The severe recurrent infections raised the possibility of an immunodeficiency, which was confirmed by flow cytometry of the white blood cells in which significantly decreased lymphocytes were detected. Although hypocalcaemic episodes and high parathyroid hormone levels were reported in the neonatal period, microdeletion of the DiGeorge region of chromosome 22 was excluded. Generalized oedema, deterioration of right ventricular function and recurrent infections progressed, the patient needed continuous mechanical ventilation and despite therapy died at the age of nine month.In search of metabolic diseases urine organic acids, serum amino acids, ammonia and serum transferrin isoelectric focusing were performed to exclude congenital disorders of glycosylation, but all yielded normal results. Routine karyotyping revealed no visible chromosomal aberration and Fluorescence In Situ Hybridization (FISH) analysis of the DiGeorge syndrome critical region (22q11.2) gave also a normal result (Figure 1).

## Methods

Array CGH was performed using the Agilent Human Genome G3 Sureprint 8x60K Microarray (Agilent Technologies, Stewens Creek Bld., Santa Clara, CA, 95051 USA), a high resolution 60-mer oligonucleotide based microarray containing 55,077 60-mer probes, spanning coding and non-coding genomic sequences with median spacing of 33 kb and 41 kb, respectively.

Array image was acquired using an Agilent laser scanner G2565CA (Agilent Technologies, California, USA) and analyzed with the Agilent Feature Extraction software (v10.10.1.1.). Results were presented by Agilent Cytogenomics software (v2.5.8.11). DNA sequence information refers to the public UCSC database (Human Genome Browser, Feb 2009 Assembly; GRCh37:hg19).

The deletion detected was aligned to known aberrations listed in publicly available databases, such as the Database of Chromosomal Imbalance and Phenotype in Humans using Ensembl Resources (DECIPHER [5]), the Database of Genomic Variants (DGV [6]), Ensembl [7] and European Cytogeneticists Association Register of Unbalanced Chromosome Aberrations (ECARUCA [8]).

Parental samples were analyzed using the same array.

## Results and discussion

**Figure 2:**
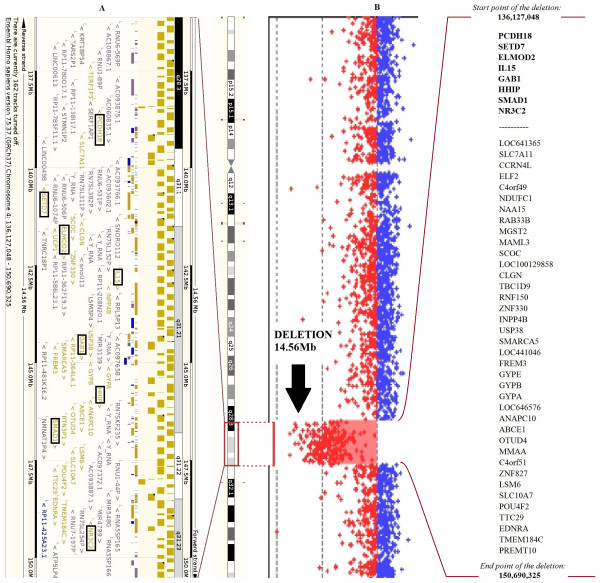
**Ensembl and aCGH image of the deleted region with the affected genes.** Part **B** is our aCGH image where the deleted region is clearly visible and its location on chromosome 4 is marked. Listed on the right side are all of the genes affected by this deletion. The first eight are emphasized because these are most likely to affect the phenotype. Part **A** is the Ensembl image of this area with the affected genes highlighted [9].

Array CGH analysis of our patient with complex malformations revealed a 14.56 Mb deletion on the long arm of chromosome 4 (4q28.3q31.23; 136,127,048 - 150,690,325) (see Figure 2). Analysis of the parents confirmed the de novo occurrence of the deletion. In a detailed analysis of the genes affected by the deletion we highlighted 8 genes (*PCDH18, SETD7, ELMOD2, IL15, GAB1, HHIP, SMAD1, NR3C2)* with possible contributions to the phenotype. The genes thought to be relevant are summarized in Table 1 in order of their position on the chromosome.

**Table 1:** Deleted genes on the long arm of the chromosome 4, with a possible effect on the phenotype

**GENES WITH POSSIBLE EFFECT ON THE PHENOTYPE**			
**Gene**	**Role**	**Position on chr 4**	**Effect on the phenotype**
*PCDH18*	Protocadherin 18: impact on cell-cell connection, highly expressed in the brain and lung.	138,440,071	Might be involved in intellectual disability [10].
		-	
		138,453,628	
*SETD7*	Histone methyltransferase H3K4.	140,427,191	Regulation of the neural genes; differentiation of neuroectoderm progenitor cells [11].
		-	
		140,477,576	
*ELMOD2*	GTPase activating protein: role in antiviral responses and pulmonary fibrosis.	141,445,311	Mutations in this gene may lead to familial idiopathic pulmonary fibrosis [12].
		-	
		141,474,923	
*IL15*	Cytokine:affects T-cell activation and proliferation, controls the number of CD8+ memory T cells.	142,557,748	Dosage alteration of IL15 gene may explain patient’s low lymphocyte number [13].
		-	
		142,655,139	
*GAB1*	Direct substrate of the epidermal growth factor receptor (EGFR).	144,257,679	Has an important role in branching tubulogenesis, and probably in the lung vessel malformation observed in our patient [14,15].
		-	
		144,359,717	
*HHIP*	Hedgehog-Interacting Protein: effect on regulation of morphogenesis.	145,567,417	Possible correlation between the lung malformation of our patient and the gene [16,17].
		-	
		145,662,541	
*SMAD1*	Involved in the downstream signaling pathway of bone morphogenic protein (BMP) subfamily members.	146,402,950	SMAD1 deficiency may play a role in the development of pulmonary hypertension [18-20].
		-	
		146,480,327	
*NR3C2*	Mineralocorticoid receptor: impact on the regulation of sodium concentration in the body. *NR3C2* mutations were published to influence the development of hyponatraemia.	148,999,914	Copy number changes of *NR3C2* gene may be associated with low sodium levels [21].
		-	
		149,365,849	

The first of these genes is *PCDH18* (MIM *608287). Kasnauskiene J. et al. reported a boy with severe developmental delay, seizures, microcephaly and hypoplastic corpus callosum with a 1.53 Mb single gene deletion on chromosome 4 (4q28.3; 137,417,338 – 138,947,282) with array CGH. Deletion of the *PCDH18* gene was also detected which together with our case suggests that the *PCDH18* gene might be involved in intellectual disability [10]. Unfortunately our patient died at the age of nine months, she was hospitalized many times and showed severe developmental delay, however we have no data about her exact intellectual ability, but it is a repeatedly described symptom in previous reports on 4q interstitial deletions where the region partially overlaps with ours. *SETD7* (*606594) gene could be a second candidate in connection with cognitive development. Wang et al. examined the expression of genes responsible for epigenetic changes in different neuronal tissues, especially during differentiation. It was determined that *SETD7* expression was upregulated both in human embryonic stem cells and human cortex, which implies that *SETD7* might play a particular role in the regulation of the neural genes [11].

The laboratory results of our patient showed decreased lymphocyte numbers which may be responsible for the patient’s immunodeficiency. Another gene of the deleted region is the *IL15* (*600554) (see Table 1). Ku et al. suggested earlier that the balance between *IL15* and *IL2* can control the number of the CD8+ memory T-cells. Dosage alteration of this gene may explain our patient’s low lymphocyte number [13].

The clinical picture of our patient was complicated by vascular lung malformation affecting both the pulmonary arteries and veins and consequent pulmonary hypertension. The *GAB1* (*604439) gene, which encodes an adaptor protein and is involved in branching tubulogenesis [14,15] may also be associated with the vascular malformation of the lung observed in our patient.

The *HHIP* (*606178) gene encodes a protein which participates in the regulation of morphogenesis. Zhou et al. found decreased levels of *HHIP* mRNA and protein in the lung tissue samples of COPD patients. Based on these, a possible correlation between the abnormal development of lung vessels in our patient and the gene can be hardly excluded [16,17].

The unique feature of vascular malformation in the lung, gradually lead to pulmonary hypertension in our patient. Additionally the role of genes in the deleted region associated with the development of pulmonary hypertension cannot be ruled out. Han et al., examined the *SMAD1* (*601595) gene (in knock-out mice), and found that the deficiency of it is associated with pulmonary hypertension [18]. Pannu et al. noticed the importance of *SMAD1* in the development of fibrosis [19]. In a publication by Nasim et al. the same results were discovered when they examined a 47-year-old French woman with primary pulmonary hypertension [20]. Furthermore, mutations in *ELMOD2* (*610196) gene (deleted in our patient) may lead to familial idiopathic pulmonary fibrosis [12], however in our patient we have no data about parenchymal changes of the lung.

Another gene affected by this deletion with possible effect on the phenotype, called *NR3C2*, plays a role in regulating the sodium concentration in the body thereby influencing fluidic balance and blood pressure. We cannot rule out the possibility that changes in copy number of this gene may be associated with low sodium levels consequently seen in our patient [21].

## Conclusion

Several genes are affected in the deletion of the region between 4q28.3 and 4q31.23, which could give a possible explanation for the clinical symptoms. Among other functions a role in lung morphogenesis and tubulogenesis can be attributed to the deleted genes in our patient which may explain the unique feature of vascular malformation of the lung vessels leading to pulmonary hypertension. The interpretation of the role of the haploinsufficient genes is complicated by the fact that in current literature we only found a few cases where the deletion overlapped our region. In the future it would be necessary to examine this region not only with array CGH but also with transcriptomic and proteomic methods.

## Consent

Because the patient died, written consent for publication and any accompanying images in this Case report was obtained from the patient’s next of kin. A copy of the written consent is available for review by the Editor-in-Chief of this journal.

## Abbreviations

Array CGH: Array comparative genome hybridization; COPD: Chronic obstructive pulmonary disease; CT: Computed tomography; DECIPHER: Database of chromosomal imbalance and phenotype in humans using ensembl resources; DGV: Database of genomic variants; ECARUCA: European cytogeneticists association register of unbalanced chromosome aberrations; EEG: Electroencephalography; FISH: Fluorescence in situ hybridization; UCSC: University of california Santa Cruz.

## Competing interests

The authors declare that they have no competing interests related to this manuscript.

## Authors’ contributions

KK and KH were responsible for the patient’s clinical genetic examination and KT was liable for the clinical and cardiologic examination. KK, KT and KH contributed to the clinical description. BM, MCz and BD conceived and designed the molecular experiments. MCz and BD performed the array CGH and analyzed the data. BD, MCz, KK, KH, GyK and BM co-wrote the manuscript. KS and PK revised the manuscript critically for important intellectual content. All authors read and approved the final manuscript.
